# Low-intensity vestibular noise stimulation improves postural symptoms in progressive supranuclear palsy

**DOI:** 10.1007/s00415-024-12419-9

**Published:** 2024-05-09

**Authors:** Max Wuehr, Daniela Peto, Urban M. Fietzek, Sabrina Katzdobler, Georg Nübling, Mirlind Zaganjori, Matthias Brendel, Johannes Levin, Günter U. Höglinger, Andreas Zwergal

**Affiliations:** 1 German Center for Vertigo and Balance Disorders (DSGZ), LMU University Hospital, LMU Munich, Marchioninistrasse 15, 81377 Munich, Germany; 2 Department of Neurology, LMU University Hospital, LMU Munich, Munich, Germany; 3 Schön Klinik München Schwabing, Munich, Germany; 4 Deutsches Zentrum Für Neurodegenerative Erkrankungen (DZNE) E.V., Munich, Germany; 5 Munich Cluster of Systems Neurology (SyNergy), Munich, Germany; 6 Department of Nuclear Medicine, LMU University Hospital, LMU Munich, Munich, Germany

**Keywords:** Progressive supranuclear palsy, Atypical parkinsonism, Galvanic vestibular stimulation, Stochastic resonance, Balance, Body sway

## Abstract

**Background:**

Postural imbalance and falls are an early disabling symptom in patients with progressive supranuclear palsy (PSP) of multifactorial origin that may involve abnormal vestibulospinal reflexes. Low-intensity noisy galvanic vestibular stimulation (nGVS) is a non-invasive treatment to normalize deficient vestibular function and attenuate imbalance in Parkinson’s disease. The presumed therapeutic mode of nGVS is stochastic resonance (SR), a mechanism by which weak sensory noise stimulation can enhance sensory information processing.

**Objective:**

To examine potential treatment effects of nGVS on postural instability in 16 patients with PSP with a clinically probable and [^18^F]PI-2620 tau-PET-positive PSP.

**Methods:**

Effects of nGVS of varying intensity (0–0.7 mA) on body sway were examined, while patients were standing with eyes closed on a posturographic force plate. We assumed a bell-shaped response curve with maximal sway reductions at intermediate nGVS intensities to be indicative of SR. An established SR-curve model was fitted on individual patient outcomes and three experienced human raters had to judge whether responses to nGVS were consistent with the exhibition of SR.

**Results:**

We found nGVS-induced reductions of body sway compatible with SR in 9 patients (56%) with optimal improvements of 31 ± 10%. In eight patients (50%), nGVS-induced sway reductions exceeded the minimal clinically important difference (improvement: 34 ± 5%), indicative of strong SR.

**Conclusion:**

nGVS yielded clinically relevant reductions in body sway compatible with the exhibition of SR in vestibular sensorimotor pathways in at least half of the assessed patients. Non-invasive vestibular noise stimulation may be thus a well-tolerated treatment strategy to ameliorate postural symptoms in PSP.

## Introduction

Progressive supranuclear palsy (PSP) is a rare and rapidly progressing neurodegenerative disease that clinically belongs to the group of atypical parkinsonian syndromes and is characterized by cerebral aggregation of tau protein [7, 53]. Early postural instability and unexplained recurrent falls are central to the clinical presentation of PSP and a diagnostic criterium to differentiate the disease from idiopathic Parkinson’s disease [24, 35]. Postural symptoms are a major disabling factor of PSP and a determinant of survival due to the associated risk of secondary injuries and immobilization [9, 42]. Balance deficits in PSP are likely of multifactorial origin and may involve axial rigidity, supranuclear gaze palsy, and deficient central sensorimotor balance regulation including abnormal vestibular balance reflexes [9, 36, 69]. As of yet, there is no disease-modifying therapy available for PSP, and existing medical and non-medical approaches to improve balance and prevent falls in patients yield moderate and transient improvements, if any [7, 9, 11, 51, 52].

A vestibular origin of postural symptoms and falls in PSP has been repeatedly discussed [5, 36, 56, 69]. There is, albeit conflicting, clinical evidence for abnormal peripheral vestibular function related to otolith pathways in PSP [5, 21, 36, 41, 56]. Vestibular deficits could be an accompanying age-related symptom, as PSP is a late-onset disease, or disease-specific directly associated with the pathophysiology of PSP. Brain imaging studies in patients with Parkinson’s disease and PSP further point to a common dysfunction within central cholinergic networks that transmit vestibular afferent inputs to the thalamus and basal ganglia, which closely correlates with postural deficits and falls in afflicted patients [6, 40, 69]. A key node within this network is the pedunculopontine nucleus (PPN) that provides excitatory cholinergic input to the thalamus and undergoes substantial neuronal loss early in the course of PSP [68]. Previous attempts using deep brain stimulation in PSP have already identified the PPN as a potential therapeutic target, however, without yielding clear and consistent benefit in tested patients [47]. A non-invasive method to modulate PPN activity is galvanic vestibular stimulation (GVS) [10]—a technique to activate vestibular afferents by weak electric current [13]. Delivered as a low-intensity and imperceptible random noise stimulus (called noisy GVS; nGVS), it has been previously shown to improve postural imbalance and a range of motor and non-motor symptoms in patients with Parkinson’s disease [34, 43, 44, 46, 55, 66, 67]. The presumed therapeutic mode underlying treatment effects of nGVS is stochastic resonance (SR)—a mechanism by which sensory information processing becomes enhanced at the presence of a particular non-zero amount of sensory noise [12, 37, 62].

**Fig. 1:**
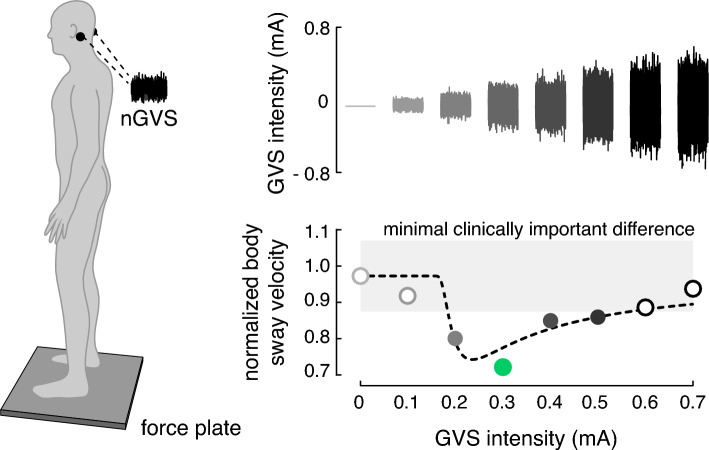
Experimental setup and procedures. Left side: Effects of noisy galvanic vestibular stimulation (nGVS) on static balance in patients were measured on a posturographic force plate. Velocity of body sway was calculated from the resultant center-of-pressure trajectories. Right side: Exemplary modulation of body sway (hypothetical data, lower panel) across the administered nGVS intensities (upper panel) that follows a bell-shaped performance curve indicative of the presence of stochastic resonance (model fit: dashed line). Filled dots indicate body sway reductions greater than the minimally important difference (grey area). The green filled dot indicates the optimal reduction of body sway at a particular nGVS level

The aim of this study was to examine potential treatment effects of vestibular neuromodulation via nGVS on postural instability in patients with PSP. It is known from previous studies that treatment effects of nGVS critically depend on the stimulation intensity. Intermediate noise intensities improve vestibular signal transfer whereas low or high stimulation intensities either not affect or even disturb signal processing [18, 38]. Accordingly, the characteristic signature indicating nGVS-induced SR and a positive treatment response is a bell-shaped performance curve, where the performance metric (i.e., postural balance) becomes optimally enhanced at a specific intermediate level of noise (Fig. 1). To identify nGVS-induced balance improvements in patients with PSP, we therefore studied postural responses to nGVS across a broad range of stimulation intensities and used different established criteria to determine whether stimulation-induced modulations of postural instability are compatible with the exhibition of SR. We further studied whether demographic or disease-related characteristics (e.g., duration of disease, severity of symptoms, or tau-deposition pattern) are associated with potential treatment responses.

## Materials and methods

### Participants

Sixteen patients (age 70.7 ± 7.8 years, 6 females) with a probable PSP according to the MDS-PSP criteria [24] participated in the study (for detailed patient characteristics, see Table 1). [^18^F]PI-2620 PET imaging was performed to depict tau-deposits as an in vivo biomarker supporting the clinical diagnosis [8]. Each patient underwent a complete physical, neurological and neuro-otological examination by an expert neurologist, including a clinical assessment of vestibuloocular (head impulse test) and vestibulospinal (Romberg’s test) function (AZ). Symptom severity was scored using the Progressive Supranuclear Palsy Rating Scale (PSPRS) [20], while patients were taking their regular medication (i.e., L-dopa at a mean daily dose of 518 ± 254 mg in 10 patients). Patients had a mild-to-moderate disease severity with a PSPRS of 29.8 ± 11.8. Fourteen age-matched healthy controls (age 68.9 ± 6.5 years, 9 females) were included in the study to establish normative data. All participants gave written informed consent prior to study inclusion.

**Table 1:** Clinical characteristics and global stimulation effects of patients

ID	Gender	Age (y)	Disease duration (y)	PSP type	Laterality	L-dopa (mg)	PSPRS	Relevant comorbidities	Exhibition of SR
P1	M	59	2	PSP-RS	Symmetric	L-dopa (300)	17	D	Strong
P2	M	78	4	PSP-RS	Left-dominant	L-dopa (800)	44	CI, D	None
P3	F	75	3	PSP-FTD	Left-dominant		37	CI	None
P4	M	80	3	PSP-P	Symmetric	L-dopa (300)	26		None
P5	M	63	4	PSP-P	Right-dominant	L-dopa (600)	20		Strong
P6	M	73	2	PSP-RS	Symmetric		26		None
P7	F	76	1	PSP-RS	Symmetric		30	CI	None
P8	M	75	3	PSP-P	Left-dominant	L-dopa (900)	45	D, PS, CC	Strong
P9	F	82	7	PSP-PAGF	Right-dominant	L-dopa (400)	34		Strong
P10	F	64	1.5	PSP-RS	Symmetric	L-dopa (600)	31	D	None
P11	M	80	5	PSP-RS	Symmetric	L-dopa (1000)	52		Strong
P12	M	61	5	PSP-RS	Symmetric		15		None
P13	F	60	4	PSP-P	Left-dominant	L-dopa (375)	19	PS	Weak
P14	F	71	3	PSP-RS	Left-dominant		33		Strong
P15	M	65	1	PSP-RS	Right-dominant		10		Strong
P16	M	69	1	PSP-RS	Left-dominant	L-dopa (400)	38		Strong

*PSP* progressive supranuclear palsy, *PSPRS* PSP rating scale, *RS* Richardson’s syndrome, *P* parkinsonism, *PAGF* pure akinesia and gait freezing, *FTD* frontotemporal dysfunction, *D* depression, *CI* cognitive impairment, *CC* camptocormia, *PS* Pisa syndrome, *SR* stochastic resonance

### PET imaging and data analysis

[^18^F]PI-2620 PET imaging was performed in a full dynamic setting (0–60 min post injection) with a Siemens Biograph or mCT PET/CT scanner (Siemens Healthineers, Erlangen, Germany) at the Department of Nuclear Medicine, LMU Munich as described earlier [8, 57]. The multilinear reference tissue model 2 (MRTM2) in PMOD version 3.9 (PMOD Inc) was used to calculate distribution volume ratio images (DVR; DVR = non-displaceable binding potential + 1) of each full dynamic data set. The cerebellum excluding the dentate nucleus and the central cerebellar white matter as well as the superior and the posterior cerebellar layers (thickness in z direction = 1.5 cm each) served as a reference region.

Two expert readers (M.B. and M.Z.) performed a dichotomous visual read of DVR maps. In addition, [^18^F]PI-2620 DVR values were obtained in the following PSP target regions predefined by Brainnetome atlas [14]: frontal lobe, thalamus, globus pallidus, and putamen (left- and right-sided, respectively). Patient data were compared to a normative in-house data set derived from a matched healthy control group of the same scanners.

### Galvanic vestibular stimulation

Vestibular noise stimulation (i.e., nGVS) was applied via a pair of 4.0 cm × 6.0 cm Ag–AgCl electrodes attached bilaterally over the left and right mastoid process. Zero-mean Gaussian white noise stimulation with a frequency range of 0–30 Hz and varying peak amplitudes of 0–0.7 mA was delivered by a mobile constant current stimulator (neuroConn^®^, Illmenau, Germany).

### Experimental procedures

Body sway was recorded for 30 s on a posturographic force plate (Kistler, 9261A, Kistler Group, Winterthur, Switzerland) at 40 Hz while patients were standing with their eyes closed (Fig. 1A). This procedure was repeated eight times, while patients were stimulated with a different amplitude of nGVS (ranging from 0 to 0.7 mA, in a randomized order) in each trial. Patients were blinded to the exact stimulation order. Between trials, patients were given a short break to recover.

### Data and statistical analysis

For each stance trial, mean sway velocity was calculated as the primary output measures based on the recorded radial center-of-pressure (CoP) trajectory using the formula $SV = 1/T \times {\sum }_{i}\left|{r}_{i+1}-{r}_{i}\right|, [{\text{mm}}/{\text{s}}]$, where $T$ is the total trial duration (i.e., 30 s) and ${r}_{i}$ is the radial CoP distance of the *i*th sample. For further analysis, sway velocity measures from the eight stance trials were normalized to sway velocity obtained during 0 mA stimulation (i.e., baseline condition).

To determine whether SR-like dynamics were present in the balance responses of patients to varying nGVS levels, we tested three increasingly rigorous criteria that built on one another: (1) The first criterion tested whether body sway of patients improved for at least one particular nGVS level compared to baseline condition (i.e., 0 mA nGVS). (2) The second criterion was based on a visual inspection of response dynamics of body sway across increasing nGVS level by three experienced human raters (i.e., MW, DP, and AZ). Each rater had to evaluate whether (in addition to the fulfillment of the first criterion) nGVS-amplitude-dependent changes of body sway in individual patients were further compatible with a bell-shaped response curve with improvements of performance at intermediate stimulation intensities that is indicative of the presence of SR. This evaluation was based on a plot of the normalized nGVS-dependent changes in body sway and a concomitant plot of a theoretical SR curve that was fit on the data using a goodness-of-fit statistic [2, 18] (Fig. 1B). The applied equation fit represents an adapted version of the originally proposed SR model by Benzi [3], including a piecewise, linear masking effect to model cases where nGVS effects at high amplitudes may have detrimental effects on the performance metric [58]. The criterion was met if at least two of the raters identified the presence of SR-like dynamics. (3) The third criterion additionally evaluated whether improvements at intermediate nGVS levels were greater than the minimal clinically important difference (MCID; defined as half the standard deviation for normative data [60]) for changes in body sway velocity. MCID for sway velocity was 2.3 mm/s calculated based on the posturographic recordings of the 14 age-matched healthy individuals standing with eyes closed for 30 s.

Based on the three criteria, patients were classified as showing solely optimal improvement and no SR (criterion 1), exhibiting weak SR (criterion 1 & 2) or showing strong SR (criterion 1 & 2 & 3). Potential correlations between SR classification and age, gender, disease duration, disease severity (i.e., PSPRS), and baseline body sway were analyzed using Spearman's rank correlation. Extent of Tau deposition in predefined regions of interest was compared between patients with weak/strong SR and no SR using multivariate analysis of variance including age and sex as covariates and Bonferroni adjustment for multiple comparisons. Descriptive statistics are reported as mean ± SD. All results were considered significant at *p* < 0.05. Statistical analysis was performed using SPSS (Version 26.0, IBM Corp., USA).

## Results

Application of nGVS at intensities ranging from 0.1 to 0.7 mA was well tolerated and did not cause apparent disequilibrium in any of the examined patients. In a first step of analysis, we evaluated whether body sway velocity was decreased by at least one particular nGVS intensity compared to sham stimulation (i.e., nGVS at 0 mA). This criterion was met by 12 patients (75%) with an optimal mean improvement magnitude of 25% (range 2–45%) at an average intensity of 0.4 mA (range 0.1–0.7 mA).

**Fig. 2:**
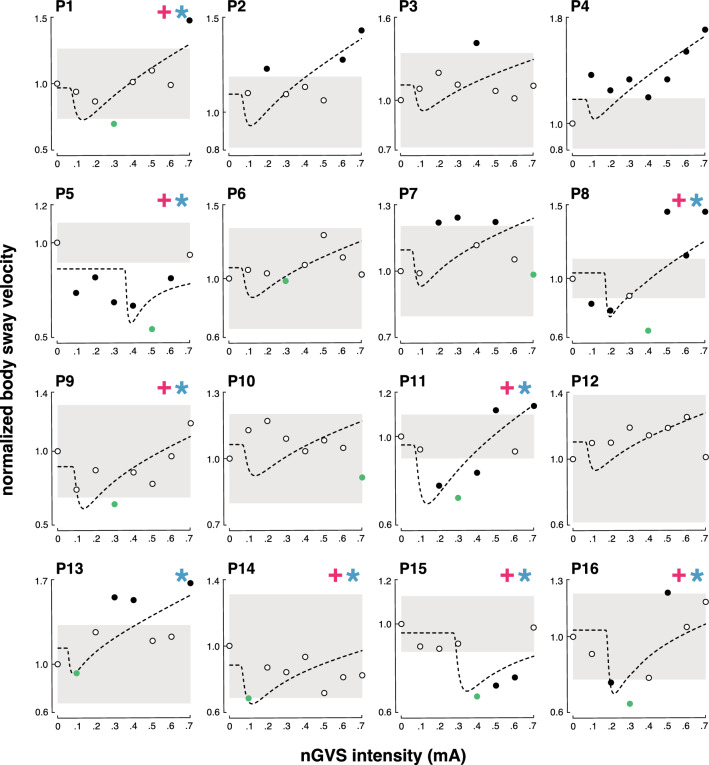
Individual effects of low-intensity vestibular noise stimulation on static balance. Normalized body sway responses to noisy galvanic vestibular stimulation (nGVS) are plotted against the administered nGVS levels for each individual patient. Dashed lines represent the stochastic resonance (SR) model fits. Black filled dots indicate body sway modulations greater than the minimally important clinical difference (grey area). Green filled dots indicate maximum reductions of body sway at particular nGVS levels. Blue asterisks denote those patients that exhibit SR-like responses according to three human judges (weak SR). Pink crosses denote those patients that additionally show clinically meaningful improvement of body sway (strong SR)

**Fig. 3:**
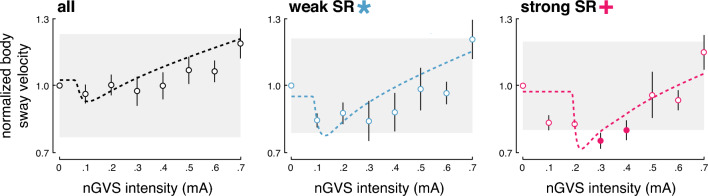
Group average effects of low-intensity vestibular noise stimulation on static balance. Group average normalized body sway responses (mean ± SEM) to noisy galvanic vestibular stimulation (nGVS) are plotted for each of the administered nGVS levels for all patients (left panel), those patients exhibiting at least weak stochastic resonance (SR; middle panel), and those exhibiting strong SR (right panel). Filled dots indicate body sway modulations greater than the minimally important clinical difference (grey area)

In a second step, an established SR model was fit to the individual modulations of body sway velocity in dependence of nGVS intensity (Fig. 2). Three experts were asked to independently rate for each patient by visual inspection of individual sway velocity modulations and corresponding model fits, whether body sway responses follow a bell-shaped performance curve or not. Based on their judgments, SR-like treatment responses to nGVS were present in nine patients (56%) with optimal improvements of 31% (range 7–45%) at an average intensity of 0.3 mA (range 0.1–0.5 mA). Analogous bell-shaped performance modulations with optimal improvement at intermediate noise intensities were found on the group average response level of these patients (Fig. 3). In the remaining patients (44%), body sway velocity either randomly fluctuated (3 patients) or was generally increased (4 patients) across the range of tested nGVS intensities.

We subsequently identified those patients that in addition to SR-like response dynamics showed a clinically meaningful improvement of static balance (i.e., a reduction of body sway velocity greater than the MCID, Fig. 2). This criterion for the exhibition of strong SR was met by 8 patients (50%) with a mean optimal improvement of 34% (range 28–45%) at an average intensity of 0.3 mA (range 0.1–0.5 mA). Considerable SR-like performance improvements were also apparent on the group average level of patients exhibiting strong SR (Fig. 3).

**Fig. 4:**
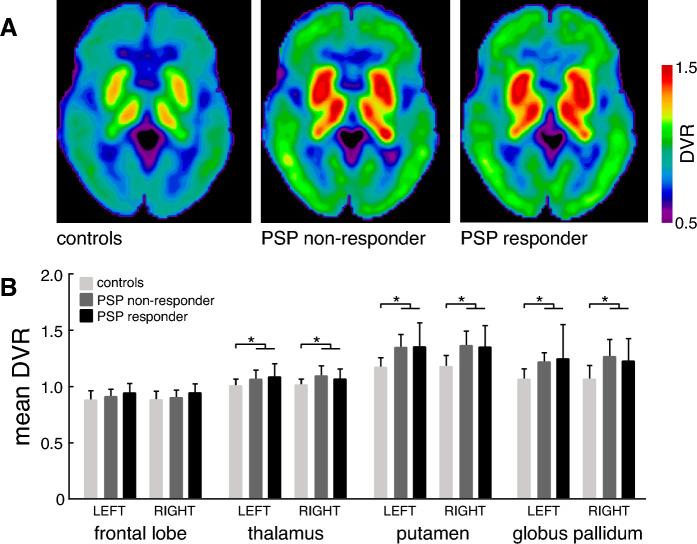
[^18^F]PI-2620 binding in PSP subgroups and controls. **A** Average [^18^F]PI-2620 distribution volume ratio (DVR) maps presented as axial overlays on a standard magnetic resonance imaging template for all study groups. Extracerebral voxels were masked. **B** [^18^F]PI-2620 DVR comparisons for selected progressive supranuclear palsy (PSP) target regions between healthy controls and patients that respond or do not respond to treatment with noisy galvanic vestibular stimulation (nGVS)

In a final step, we explored demographic and disease-related factors that may potentially promote or hamper the exhibition of weak or strong SR in response to nGVS treatment. We did not find any correlation between age, gender, disease duration, PSPRS, or body sway at baseline and nGVS treatment responses of individual patients. In addition, mean DVR in the left and right frontal lobe, thalamus, putamen, and globus pallidus were not statistically different for patients with and without SR. Expectedly, whole-brain DVR and mean DVR in the bilateral thalamus, putamen, and globus pallidus were significantly higher in both PSP subgroups compared to the control group (Fig. 4).

## Discussion

In this study, we examined the effects of a weak vestibular neuromodulation by nGVS on postural instability in patients with PSP. The purported mode of action of nGVS is SR, by which sensory-(motor) processing can become enhanced at the presence of low-intensity additive sensory noise below the sensory detection threshold [12, 37, 62]. In half of the assessed patients with PSP, we found robust stimulation responses compatible with SR that were linked to clinically meaningful improvements in static balance regulation, i.e., 28–45% reduction in sway velocity—a measure that has been closely linked to the frequency of falls in afflicted patients [69]. The observed response rate to treatment is higher compared to healthy individuals, where nGVS-induced balance responses compatible with SR were only rarely reported [2]. Consistent with previous reports [18, 66], the average nGVS intensity that optimally improved postural sway was found at 0.3 mA, which corresponds to approximately 60% of the estimated detection threshold of vestibular afferent responses to GVS [33]. Previous studies provide accumulating evidence that nGVS can attenuate postural imbalance and other motor and autonomic symptoms in patients with Parkinson’s disease [34, 43, 44, 46, 55, 66, 67]. To our knowledge, this is the first study suggesting that nGVS treatment effects may extend to postural symptoms in patients with PSP.

Multifaceted reasons for postural imbalance and falls in PSP are currently discussed that point to at least two distinct mechanisms of action by which nGVS could impact postural symptoms in afflicted patients. First, there is, however not uncontroversial, evidence that links disequilibrium and falls in PSP to vestibular dysfunction [5, 21, 36, 41, 56]. Accordingly, the previous reports suggest that in particular otolith-mediated ocular-motor and balance reflexes can be impaired in patients with PSP [5, 36, 56]. In this context, nGVS-induced facilitation of vestibular signal processing has been shown to not only sensitize vestibular perception as such [18, 31, 32, 63], but to also enhance the responsiveness of vestibulospinal balance reflexes in healthy individuals and patients with chronic vestibular hypofunction [48, 61]. Improved vestibulospinal function by nGVS has further been associated with a stabilization of balance during standing and walking under vestibular-challenging circumstances in both cohorts [15, 17, 19, 25–28, 39, 64, 65]. Hence, nGVS could attenuate postural imbalance in PSP by restoring deficient vestibular-related balance responses in afflicted patients.

Alternatively, nGVS treatment in PSP could take effect along ascending pathways that connect vestibular afferents to the thalamus and basal ganglia [10, 54]. The PPN, a central neuronal hub along this pathway, provides the primary cholinergic input to the thalamus. Natural vestibular input as well as vestibular neuromodulation via nGVS were shown to directly modulate PPN activity in animal models and humans [1, 10, 49]. In PSP, neuronal loss in the PPN and the thalamus is a common and early neurodegenerative sign in the course of disease [22, 29, 59, 68], which entails a substantial reduction of thalamic cholinergic activity in afflicted patients [23]. PSP-associated neurodegeneration within this mesencephalic brainstem–thalamus loop appears to be further closely associated with patients’ postural imbalance and frequency of falling as suggested by functional brain imaging [9, 69]. Hence, nGVS may attenuate postural instability in PSP via an activation of tegmental brainstem nuclei that restores excitatory cholinergic input to the thalamus.

It is eventually also conceivable that nGVS treatment effects on posture in PSP may be rather unrelated to the primary PSP pathophysiology. The average age of disease onset in PSP is in the sixth decade of life [45] corresponding to mean disease onset at 67 years (range 49–77 years) in our cohort of patients. Vestibular sensitive capacity and related motor functions are known to gradually decline above the age of 40 [4]. A close association between postural imbalance and age-related decline in vestibular–perceptual thresholds has been previously reported [30]. This may explain why beneficial responses of vestibular perceptual and sensorimotor function to nGVS treatment become more frequent and pronounced in the elderly compared to the young healthy population [16, 25, 50]. Age rather than disease-related impairments in vestibular balance control could therefore also be the source of nGVS-induced balance improvements in patients with PSP. The latter assumption is supported by the lack of correlation between treatment responses and disease duration, disease severity scores, or the extent of tau load in PSP target regions derived from [^18^F]PI-2620 PET.

In summary, we found that vestibular neuromodulation via nGVS yielded clinically meaningful reductions of postural instability in half of the assessed patients with PSP. Long-term application of nGVS in patients has previously shown to be safe with negligible side effects. Non-invasive vestibular noise stimulation in PSP may therefore be a well-tolerated treatment option to attenuate postural symptoms, reduce the risk of falling, and preserve mobility in afflicted patients. A re-evaluation of the observed effects in a larger sample of patients is required to clarify whether and how the treatment response may depend on clinical characteristics of patients, including instrumented assessment of vestibular semicircular canal and otolith function, and whether long-term treatment in respondent patients can effectively facilitate mobility and reduce their risk of falling.

## Data Availability

The datasets used and/or analyzed during the current study will be available from the corresponding author upon reasonable request.
